# Feline caudal vena cava to aorta ratio reference interval

**DOI:** 10.1177/1098612X241303309

**Published:** 2024-12-20

**Authors:** Adam Whitelock, Wendy Goodwin, Shaun Pratt, Katherine Nash

**Affiliations:** School of Veterinary Science, The University of Queensland, Gatton, QLD, Australia

**Keywords:** Ultrasonography, caudal vena cava, aorta, reference interval, fluid assessment

## Abstract

**Objectives:**

The primary objective of this investigation was to ultrasonographically evaluate the caudal vena cava to aorta (CVC:Ao) ratio in healthy, conscious cats and to generate reference intervals. A secondary objective was to identify the site of examination with the least intra- and inter-observer variability. This investigation was undertaken to assess whether the CVC:Ao ratio holds promise as a technique to assess intravascular volume responsiveness in cats.

**Methods:**

In total, 42 healthy cats were included for reference interval generation. Ultrasound examinations were performed by two operators with each examination performed twice by each operator on the same occasion. Examinations were performed on conscious cats in left lateral recumbency. Ultrasound sites investigated were the subxiphoid, hepatic intercostal, hepatorenal and iliac bifurcation. Operators also assessed each site for ‘ease of visualisation’ on a scale of 0–3.

**Results:**

Reference intervals were generated for the CVC:Ao ratio at all four ultrasonographic sites. While each site demonstrated low variability around its mean ratio, all sites exhibited significant intra- and inter-observer variability. The hepatorenal and iliac bifurcation sites were found to be the easiest to visualise (score 3; well-defined visualisation of both vessels) and had reference intervals of 0.8–1.41 and 0.75–1.2, respectively.

**Conclusions and relevance:**

The ultrasonographic assessment of the CVC:Ao ratio was possible at four anatomical locations in the cat. The hepatorenal and iliac bifurcation may offer more readily assessable CVC:Ao ratios. Further studies are necessary to assess the utility of the CVC:Ao ratio in disease states, including in hypovolaemia and hypervolaemia.

## Introduction

Feline patients often present with atypical circulatory shock patterns that complicate the recognition of shock and its likely cause. Consequently, this can result in delayed or inappropriate treatment. Feline circulatory shock states are commonly characterised by bradycardia, hypothermia and hypotension, regardless of the cause.^1,2^ Unlike in people and dogs, the indications for and responses to rapid intravenous volume resuscitation in circulatory shock states are unclear in cats.^1^ Occult heart disease also frequently occurs in cats, potentially putting them at an increased risk of volume overload when treated with fluid resuscitation.^3,4^ Consequently, veterinarians often struggle to determine the ideal treatment for critically ill cats.

In human and veterinary medicine, numerous techniques have been developed in an attempt to accurately determine circulating volume and predict fluid responsiveness. These techniques range from invasive measures requiring general anaesthesia and mechanical ventilation, to non-invasive ultrasonographic measures in conscious patients.^5–7^ The development of point-of-care ultrasound (POCUS) and ultrasonographic techniques assessing heart–lung interactions have become mainstays of the assessment of fluid status in human intensive care.^8,9^ For example, the ratio between the inferior vena cava (IVC) and the aorta (Ao), as measured by ultrasound, has shown promise in human paediatric patients for the assessment of volume status and fluid responsiveness.^10–12^ The rationale for the measurement of the vena cava is rooted in the highly compliant nature of the vessel, which leads to alterations in diameter with changes in blood volume, and a potential relationship to venous return.^13^ Assessing the vena cava in ratio with the aorta allows for normalisation of findings accounting for considerable differences in patient size. This ratio has been shown to be both sensitive and specific in determining hypovolaemia in human paediatric patients.^11,14^ These techniques are growing in popularity in veterinary medicine, although the evidence for their use is less substantial than in human medicine. In dogs, the caudal vena cava (CVC) is used as the equivalent of the IVC and, in ratio with the aorta (CVC:Ao), has been found to correlate with systolic blood pressure variations and to trend with blood volume depletion.^15,16^

With regards to ultrasonographic location, CVC:Ao ratios have been reported for people and dogs at multiple different anatomical locations, including the subxiphoid, hepatic intercostal, hepatorenal and iliac bifurcation regions.^17–21^ Unfortunately, few studies concurrently report inter-observer comparisons for the various anatomical locations. In cats, only CVC measures have been investigated. Measurement of the CVC in solitude does not account for the considerable size variation that exists between feline patients, making interpretation of results difficult. The CVC:Ao ratio has not been reported in cats.

Determination of a reference interval at an optimal ultrasonographic location will allow the incorporation of the CVC:Ao ratio into the POCUS assessment of the emergent feline patient. This technique has the potential to offer a non-invasive and rapid method of intravascular volume assessment in cats and may aid in the assessment of volume responsiveness in this species. The aim of this investigation is to report CVC:Ao reference intervals for four ultrasonographic image acquisition sites in cats. Secondary objectives include identifying the ultrasonographic sites with the least intra- and inter-observer variability and to assess the ease of image acquisition at each location.

## Materials and methods

Healthy, privately or university owned cats were enrolled between October 2022 and October 2023. Cats were included in the study if they met the following inclusion criteria: >16 weeks of age, normal physical examination, normal complete blood count and normal biochemistry. Cats also underwent a focused cardiac assessment and were included if they had a normal left atrial to aortic root ratio and left ventricular posterior wall thickness at end diastole.^22^ Cats with a history of significant ongoing disease or who became distressed during gentle restraint for ultrasound or phlebotomy were excluded. All animals were enrolled with written owner consent and study protocols were approved by the University of Queensland Animal Ethics Committee (2022/AE000408).

Ultrasound examination was performed by two operators. Operator one was a small animal emergency and critical care resident with experience and postgraduate training in POCUS, while operator two was an anaesthesia and analgesia resident with limited ultrasonographic experience and no additional training. Ultrasound examinations were performed with a microconvex 4.5–8 MHz probe (Mindray Z5, Ultramedix apogee 1000V; Mindray). Cats were briefly restrained, without sedation, in left lateral recumbency to allow for ultrasonographic evaluation of CVC and Ao diameters at four different sites: the subxiphoid, hepatic intercostal, hepatorenal and iliac bifurcation. Cats were not clipped and ultrasound contact was achieved with alcohol-based solutions. Site and operator order were randomised for each cat.

**Figure 1:**
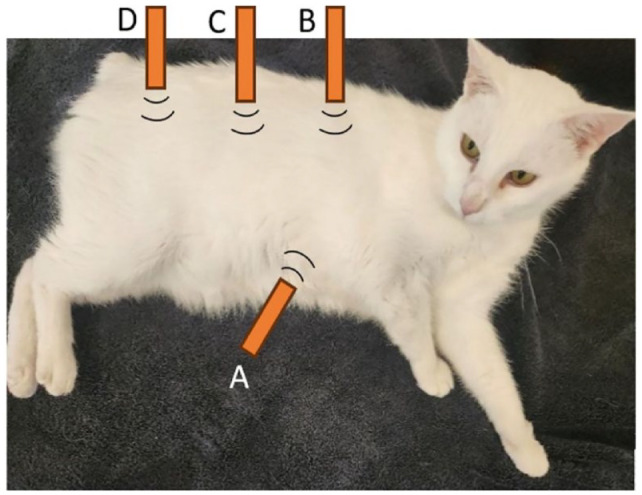
Diagrammatic representation of the investigated ultrasound sites for measurement of the caudal vena cava to aorta ratios in healthy, conscious cats: A = subxiphoid, B = hepatic intercostal, C = hepatorenal and D = iliac bifurcation

**Figure 2:**
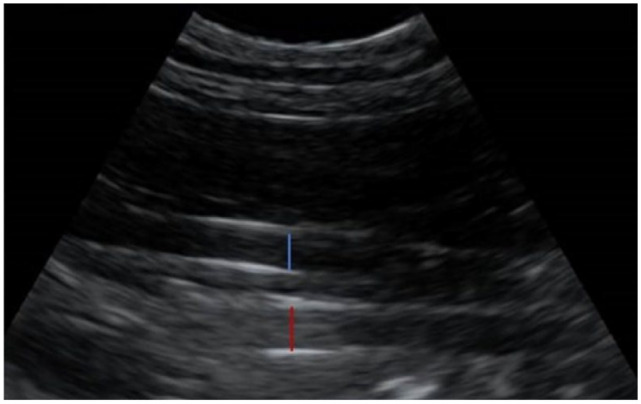
Image of the caudal vena cava and aorta at the hepatorenal site in a cat. The caudal vena cava is the vessel closest to the transducer (blue calliper), while the aorta is deep to the caudal vena cava (red calliper)

The subxiphoid view was obtained by placing the ultrasound transducer caudal to the xiphoid process in a longitudinal plane and angling it cranially to allow identification of the CVC as it crossed the diaphragm. The probe was then fanned laterally to visualise the Ao and CVC simultaneously. The hepatic intercostal window was identified by placing the transducer longitudinally on the dorsal aspect of intercostal spaces 9–11. The probe was then fanned in a dorsoventral plane until both the CVC and Ao were viewed. The hepatorenal view was taken at a standardised POCUS location.^23^ It was obtained by placing the probe longitudinally on the dorsal third of the lateral aspect of the right abdomen. The probe was then fanned dorsoventrally until the CVC and Ao were viewed caudal to the right kidney. The iliac bifurcation was imaged by placing the transducer on the lateral aspect of the right abdomen and fanning until the CVC and Ao were viewed. The vessels were then traced caudally until the bifurcation of the abdominal Ao was noted. Probe placement for each ultrasonographic site is demonstrated in Figure 1. Perpendicular diameters of each vessel were measured in B-mode on a frozen image measuring from the internal aspect of each vessel (Figure 2). Measurements were taken twice at each site during a single ultrasound. During measurement, the observer had to remove the probe from the cat before replacing it and taking the second measurement. This ultrasound protocol was then repeated by the second observer on the same day. A previously published ease-of-visualisation score was assigned for the first image acquired by each observer per site, per subject. Scores were allocated as: 0 – poor visualisation, 1 – both vessels unclear, 2 – margins of ventral vessel unclear, 3 – well defined visualisation of both vessels.^24^

All analyses were performed using MedCalc, version 22.020 (MedCalc Software). One measurement out of the four obtained per cat was randomly selected for inclusion in the reference interval calculation. Descriptive statistics, including mean, median, minimum, maximum, coefficients of skewness and kurtosis, were calculated for each view. Skewness and kurtosis values were tested if significantly different than 0.^25^ Reference intervals were calculated using the robust method recommended for sample sizes >40 and <120 by the American Society of Veterinary Clinical Pathology reference interval guidelines with 90% confidence intervals (CIs) estimated using bootstrapping.^26^ Data from skewed distributions were Box-Cox transformed prior to calculating reference intervals. Outliers were flagged using the method of Tukey fences. All flagged values were reviewed and retained for calculation of reference intervals. Intra-class correlation coefficients (ICCs) from a two-way random model and absolute agreement were calculated for inter- and intra-observer agreement. Inter- and intra-observer SD values and coefficient of variation (CV) values were calculated using the within-subject standard deviation method. ICCs were considered poor if <0.5, good if 0.5–0.75 and excellent if >0.75.

## Results

**Figure 3:**
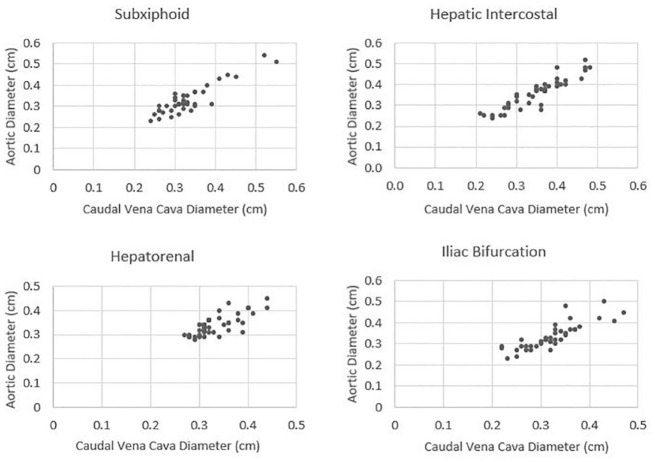
Scatterplots demonstrating caudal vena cava to aorta data spread at the subxiphoid, hepatic intercostal, hepatorenal and iliac bifurcation ultrasound sites in 42 healthy cats. Plots were measures generated from the first ultrasound of observer one for each cat

In total, 49 cats were initially enrolled and 42 cats were included for final reference interval generation and analysis. Of the seven excluded cats, four were excluded for behavioural reasons, two for abnormal focused cardiac ultrasound assessment and one due to an eosinophilia and hepatopathy on screening bloodwork. Twenty cats were female and 22 male (all desexed) and the median (range) age and weight was 3 years (1–11) and 4.3 kg (2.6–7.6), respectively. The median heart rate was 160 bpm (range 120–240). Ultrasonographic measures of the CVC and Ao were successfully obtained in all cats, by both operators. Descriptive variables and reference intervals for the CVC:Ao ratio at each ultrasound site are shown in Table 1. Of note, the hepatorenal and iliac bifurcation sites were assessed as the easiest to image as per the ‘ease-of-visualisation’ scores (Table 1). Figure 3 demonstrates the spread of the data for individual cats.

**Table 1:** Descriptive statistics and reference intervals for the caudal vena cava to aorta ratio from the subxiphoid, hepatic intercostal, hepatorenal and iliac bifurcation ultrasound sites in 42 healthy cats

Sites	Ease-of-visualisation score^*^		Mean caudal vena cava:aorta ratio	SD	Lower reference (90% CI)	Upper reference (90% CI)
	Mean	Median (range)				
Subxiphoid	2	2 (1–3)	0.99	0.103	0.72 (0.61–0.81)	1.16 (1.12–1.20)
Hepatic Intercostal	2	2 (1–3)	0.97	0.095	0.77 (0.73–0.81)	1.16 (1.11–1.20)
Hepatorenal	3	3 (2–3)	1.0	0.143	0.80 (0.76–0.83)	1.41 (1.23–1.56)
Iliac bifurcation	3	3 (2–3)	0.97	0.109	0.75 (0.70–0.81)	1.20 (1.13–1.25)

* The mean and median (range) ease-of-visualisation score is produced from the ease-of-visualisation scores from observer one, ultrasound one CI = confidence interval

When assessing intra-observer and inter-observer variability, all ICCs were <0.5 indicating no to poor inter- and intra-observer agreement. Wide CIs were also reported (most CI widths were in the range 0.5–0.6) resulting in large uncertainty in ICC estimates. As two sets of measurements were available per observer, this allowed multiple rounds of ICCs to be generated (Table 2); however, further analysis revealed very low CVs for both intra-observer and inter-observer values. The low SD and CV values demonstrate very low variation from the mean, both within the same observer and between observers.

**Table 2:** Inter-observer (observer 1 vs 2) and intraobserver (round 1 vs 2) agreement of the caudal vena cava to aorta ratio measured via ultrasound at the subxiphoid, hepatic intercostal, hepatorenal and iliac bifurcation in 42 healthy cats, taken twice (round 1 and round 2) by two different observers (observer 1 and observer 2)

View	Agreement	ICC	95% CI	SD (CV)
Subxiphoid	Observer 1 vs 2 (round 1)	−0.06	(−0.34 to 0.24)	0.10 (10%)
	Observer 1 vs 2 (round 2)	−0.15	(−0.41 to 0.14)	0.12 (12%)
	Round 1 vs 2 (observer 1)	−0.05	(−0.35 to 0.26)	0.11 (11%)
	Round 1 vs 2 (observer 2)	0.31	(0.00–0.56)	0.08 (8%)
Hepatic intercostal	Observer 1 vs 2 (round 1)	−0.16	(−0.45 to 0.16)	0.10 (10%)
	Observer 1 vs 2 (round 2)	0.06	(−0.25 to 0.35)	0.10 (10%)
	Round 1 vs 2 (observer 1)	0.43	(0.15–0.65)	0.07 (8%)
	Round 1 vs 2 (observer 2)	0.02	(−0.30 to 0.32)	0.10 (10%)
Hepatorenal	Observer 1 vs 2 (round 1)	0.22	(−0.05 to 0.48)	0.10 (10%)
	Observer 1 vs 2 (round 2)	0.23	(−0.08 to 0.50)	0.09 (9%)
	Round 1 vs 2 (observer 1)	−0.07	(−0.37 to 0.24)	0.10 (11%)
	Round 1 vs 2 (observer 2)	0.10	(−0.17 to 0.37)	0.11 (11%)
Iliac bifurcation	Observer 1 vs 2 (round 1)	−0.10	(−0.38 to 0.20)	0.10 (10%)
	Observer 1 vs 2 (round 2)	0.35	(0.07–0.59)	0.08 (9%)
	Round 1 vs 2 (observer 1)	0.31	(0.00–0.56)	0.09 (9%)
	Round 1 vs 2 (observer 2)	0.18	(−0.14 to 0.46)	0.08 (8%)

CI = confidence interval; CV = coefficient of variation; ICC = intra-class correlation coefficient

## Discussion

This study is the first to establish reference intervals for the CVC:Ao ratio in healthy cats at four distinct anatomical locations: the hepatorenal, subxiphoid, iliac bifurcation and hepatic intercostal ultrasound acquisition sites. The study design and selected ultrasound sites enabled the visualisation of both vessels in all cats, by both operators. All four locations had similar reference intervals, with the exception of the upper reference limit for the hepatorenal site which was notably larger than the other sites (Table 1). The reason for this is unclear; however, it is thought unlikely that vessel diameters vary greatly at the hepatorenal site and not at all other locations within the same population. As this is the first work comparing feline CVC:Ao ratios at various sites, an actual increased variability in vessel size at this site cannot be completely excluded and should be corroborated by additional research.

The hepatorenal and iliac bifurcation sites had greater ease-of-visualisation scores than either the subxiphoid or hepatic intercostal sites. Both the hepatorenal and iliac bifurcation sites had mean and median scores of 3, meaning that both vessel walls were well-defined (Table 1). The subxiphoid and hepatic intercostal sites had mean and median scores of 2, indicating unclear margins of the ventral vessel, hindering accurate measurement. A potential explanation for this is that there is less overlying abdominal viscera present at the hepatorenal and iliac bifurcation sites, thus allowing for greater ease of vessel visualisation.

Unfortunately, all ultrasound sites were found to have poor intra- and interobserver agreement, as indicated by ICCs <0.5. The cause for this lack of agreement is not immediately obvious. The study was designed such that each measure involved removing the ultrasound transducer from the cat before replacing it and refinding the vessels to be measured. In this way, it was hoped to limit any artificial correlation that may occur if each observer was to generate a single still image and repeat vessel measures based on this single image. This technique was also chosen to mimic finding each site independently such as may occur in clinical practice. Cats have much smaller vessels than dogs or humans and consequently, very minor inconsistencies in vessel measurement may result in large variation in CVC:Ao ratios.

In an attempt to better understand the poor agreement, further analysis of SDs and CVs of intra- and interobserver values was performed. This analysis demonstrated very limited variability of values around the mean for both intra- and interobserver values. ICC values represent the inter- or intra-observer variability relative to the total amount of variability including variability between subjects. If the amount of variability between subjects is small, then inter- and intraobserver variability that is also small in absolute magnitude may still be large relatively resulting in a poor ICC value. In this case, all between-subject SDs were in the range 0.09–0.12 (CVs = 9–11%) and all inter- and intra-observer SDs were similarly in the range 0.09–0.12 (CV = 9–12%). These levels of limited variability are likely the cause of the poor ICC values rather than a true poor agreement. Most inter- and intra-observer SD values fall close to 0.1 with CVs close to 10% suggesting good agreement. However, operators should assess whether this variability will alter clinical decision making. All sites had similar agreement when assessing via SDs; however, agreement based on ICCs was slightly stronger for the iliac bifurcation than for other sites. This may suggest a greater utility of using the iliac bifurcation as the preferred ultrasound site in clinical situations.

Poor inter- and intra-observer agreement has plagued ultrasonographic measures of volume status and fluid responsiveness in human and veterinary studies.^27,28^ In a study of 110 healthy cats, Hultman et al^29^ found only moderate intra- and inter-observer agreement of CVC diameters and poor intra- and inter-observer agreement when considering the CVC collapsibility index. Donati et al^30^ demonstrated poor intra- and inter-observer agreement of CVC collapsibility in 24 cats presenting with hypoperfusion when assessing fluid responsiveness. While agreement in our study appears stronger when assessed via CVs, these results underscore the need for caution when applying ultrasonographic measures of fluid responsiveness or volume assessment as a sole diagnostic. These techniques should always be utilised in conjunction with physical examination findings and clinical reasoning. It should also be emphasised that the present study was undertaken on clinically healthy cats. It is unclear whether our findings would remain true for cats suffering from hypovolaemia or hypervolaemia. It may be possible that the CVC:Ao ratio holds greater reliability in disease states. The CVC:Ao ratio requires validation in disease states to determine its clinical utility as a measure of volume status and fluid responsiveness.

The present study has several limitations. Foremost of these limitations is that the cats in this study were healthy. This work does not allow extrapolation of this method to cats of altered volume status. Further work with cats suffering hypo- or hypervolaemia is required to determine clinical utility. The study was undertaken in conscious cats; however, handling these unsedated cats may have induced systemic hypertension despite acclimation. In dogs, systemic hypertension has been found to decrease the CVC:Ao ratio.^31^ Because arterial blood pressure was not measured in this study, we are unable to comment further. Additionally, cats were included based on the results of a comprehensive blood analysis, physical examination and point-of-care cardiac ultrasound. Ideally, more stringent cardiovascular screening with specialist cardiologist echocardiography should have been conducted; however, this was impractical due to resource constraints. It is possible that cats with mild cardiac disease were missed in our screening and unknowingly enrolled in this study, ultimately affecting the results. Finally, alcohol-based solutions were used to improve ultrasonographic contact, but cats were not clipped for ultrasonography. This closely mimics common practice in the evaluation of clinical patients but may have reduced ultrasound acuity, impacting vessel visualisation and measurement.

## Conclusions

Assessment of the CVC:Ao ratio is achievable at the subxiphoid, hepatic intercostal, iliac bifurcation and hepatorenal sites in healthy non-sedated cats. This study defines reference intervals for these ultrasonographic locations. Intra- and inter-observer agreement varied across all sites, and further large-scale studies are needed to clarify these differences. However, the hepatorenal and iliac bifurcation sites were found to be the most technically achievable. Further studies are required to define the utility of this measure in hypovolaemic or hypervolaemic cats. If found to be indicative of volume status in disease states, this measure may offer an accessible, rapid and non-invasive technique for the assessment of critically ill cats and help determine patients that may benefit from intravascular volume expansion, with the potential for reduced morbidity and mortality in feline shock patients.
